# Amino Acid Metabolism in Rheumatoid Arthritis: Friend or Foe?

**DOI:** 10.3390/biom10091280

**Published:** 2020-09-04

**Authors:** Eleonora Panfili, Roberto Gerli, Ursula Grohmann, Maria Teresa Pallotta

**Affiliations:** 1 Department of Experimental Medicine, University of Perugia, 06132 Perugia, Italy; eleonora.panfili@unipg.it; 2 Department of Medicine, University of Perugia, 06132 Perugia, Italy; roberto.gerli@unipg.it

**Keywords:** rheumatoid arthritis (RA), tryptophan metabolism, arginine metabolism, indoleamine 2,3-dioxygenase 1 (IDO1), arginase 1 (ARG1), nitric oxide synthase (NOS), polyamines, aryl hydrocarbon receptor (AhR)

## Abstract

In mammals, amino acid metabolism has evolved to act as a critical regulator of innate and adaptive immune responses. Rheumatoid arthritis (RA) is the most common form of inflammatory arthropathy sustained by autoimmune responses. We examine here the current knowledge of tryptophan and arginine metabolisms and the main immunoregulatory pathways in amino acid catabolism, in both RA patients and experimental models of arthritis. We found that l-tryptophan (Trp) metabolism and, in particular, the kynurenine pathway would exert protective effects in all experimental models and in some, but not all, RA patients, possibly due to single nucleotide polymorphisms in the gene coding for indoleamine 2,3-dioxygenase 1 (IDO1; the enzyme catalyzing the rate-limiting step of the kynurenine pathway). The function, i.e., either protective or pathogenetic, of the l-arginine (Arg) metabolism in RA was less clear. In fact, although immunoregulatory arginase 1 (ARG1) was highly induced at the synovial level in RA patients, its true functional role is still unknown, possibly because of few available preclinical data. Therefore, our analysis would indicate that amino acid metabolism represents a fruitful area of research for new drug targets for a more effective and safe therapy of RA and that further studies are demanding to pursue such an important objective.

## 1. Introduction

Autoimmune diseases are heterogeneous conditions that involve the breakdown of tolerogenic circuitries and consequent activation of autoreactive immune cells. Rheumatoid arthritis (RA) is a chronic autoimmune disease that primarily affects joints and leads to chronic inflammation in synovium, synovial hyperplasia, and consequent joint and bone destruction. In addition, RA can result in inflammation in the lungs, pericardium, pleura, and sclera. Although the etiology of RA has not been fully understood yet, a combination of genetic and environmental factors appears to be implicated [1]. The initial disease stage of RA is associated with alterations of the immune system with consequent production of autoantibodies, targeting various molecules, including modified self-epitopes. In the following stages of the disease, both innate (i.e., dendritic cells (DCs), macrophages, and neutrophils) and adaptive immune cells (B and T lymphocytes) contribute to the amplification and perpetuation of the chronic inflammatory state. The recognition of key mediators and mechanisms implicated in the pathogenesis of RA could provide the basis for the development of new disease-modifying anti-rheumatic drugs (DMARDs) [2].

A fundamental abnormality in RA is the inappropriate growth of immune cells and stromal cells, imposing high metabolic demands to generate energy and biosynthetic precursors. Accumulating evidence suggests an important link between chronic inflammatory/autoimmune diseases, including RA [3], and ‘immunometabolism’ [4], an emerging field of investigation at the interface between the historically distinct disciplines of immunology and metabolism. Examples for metabolic changes in immunocytes in autoimmunity include modifications in cholesterol levels, glucose, and/or amino acid metabolism.

Over the evolution, some amino acid metabolic pathways have indeed become critical checkpoints for controlling adaptive immune responses to self and exaggerated inflammatory outcomes [5,6,7]. These immunoregulatory effects rely on the depletion of specific amino acids in the microenvironment and/or the generation of biologically active metabolites. Each degradative pathway is characterized by a rate-limiting enzyme, whose expression is normally subjected to strict regulation. The most important enzymes are indoleamine 2,3-dioxygenase 1 (IDO1) and arginase 1 (ARG1), which limit the catalytic rate in l-tryptophan (Trp) and l-arginine (Arg) metabolisms [8], respectively, and ornithine decarboxylase 1 (ODC1; i.e., an enzyme downstream to the ARG1-mediated pathway), which produces polyamines. An important source of amino acid metabolites (not always produced by mammals) is also the microbiota, consisting of a wide variety of bacteria, viruses, fungi, and other microorganisms that inhabit the human body in health and disease [9]. In fact, gut bacteria produce Trp metabolites that attenuate inflammation in the host [10,11] and can directly absorb amino acids and thus limit their availability to the host organism. Because an altered composition of gut microbiota has been observed in RA patients [12], the “co-metabolism” of amino acids by symbiotic microorganisms and the host may also be subjected to relevant modifications and pathogenetic consequences.

## 2. Tryptophan Metabolism and RA

Trp, the rarest essential amino acid, is a precursor for protein synthesis and the generation of several molecules involved in fundamental biological processes [5,7]. The majority of Trp (~99%) is metabolized along the kynurenine pathway and the remaining 1% is converted into serotonin and melatonin along the serotonin pathway.

### 2.1. The Kynurenine Pathway

**Figure 1:**
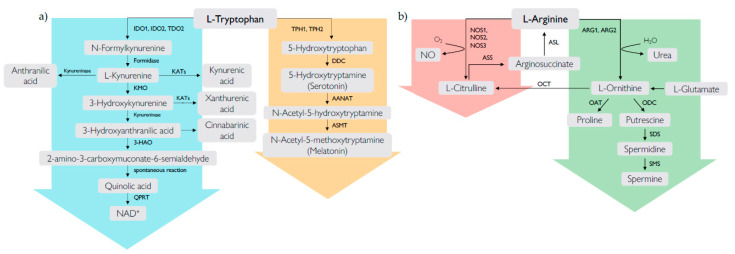
Metabolic pathways of l-tryptophan (**a**) and l-arginine (**b**). (**a**) l-Tryptophan is metabolized by two main pathways, i.e., the kynurenine pathway and the serotonin pathway. The kynurenine pathway (light blue arrow) converts approximately the 99% of ingested l-tryptophan and starts with the transformation of l-tryptophan into *N*-formylkynurenine by the enzymes indoleamine 2,3-dioxygenase 1 (IDO1), indoleamine 2,3-dioxygenase 2 (IDO2), and tryptophan 2,3-dioxygenase (TDO2). *N*-Formylkynurenine is rapidly degraded by formamidase to yield l-kynurenine, which is then converted into kynurenic acid, 3-hydroxykynurenine, or anthranilic acid via kynurenine aminotransferase (KATs), kynurenine 3-monooxygenase (KMO), and kynureninase, respectively. 3-hydroxykynurenine is converted into 3-hydroxyanthranilic acid, which is in turn metabolized by 3-hydroxyamino oxidase (3-HAO) into 2-amino-3-carboxymuconate-6-semialdehyde. This metabolite can spontaneously rearrange to form quinolinic acid (QUIN), which is used for the synthesis of nicotinamide adenindinucleotide (NAD^+^) via quinolinate phosphoribosyltransferase (QPRT). Additional lateral branches of the kynurenine pathway lead to the formation of other terminal products, including xanthurenic acid and cinnabarinic acid. The serotonin pathway (orange arrow) converts approximately the 1% of ingested l-tryptophan and starts with the transformation of l-tryptophan into 5-hydroxytryptophan by two isoforms of the enzyme tryptophan hydroxylase (TPH1 and TPH2). 5-Hydroxytryptophan is sequentially converted into 5-hydroxy-tryptamine (serotonin), N-acetyl-5-hydroxy-tryptamine (N-acetylserotonin), and N-acetyl-5-methoxytryptamine (melatonin) by the enzymes aromatic amino acid decarboxylase (DDC), arylalkylamine N-acetyltransferase (AANAT), and N-acetylserotonin O-methyltransferase (ASMT), respectively. (**b**) l-Arginine is degraded by two major families of enzymes, i.e., nitric oxide synthases (NOS) and arginases (ARG). NOS exists in three isoforms (NOS1, NOS2, NOS3) and catalyzes the conversion of l-arginine into NO and l-citrulline, which is recycled back into l-arginine by the sequential action of argininosuccinate lyase (ASL) and argininosuccinate synthetase (ASS) (pink arrow). ARG exists in two isoforms (ARG1 and ARG2) and catalyzes the degradation of l-arginine into l-ornithine and urea. l- Ornithine acts as a substrate of ornithine decarboxylase (ODC), ornithine aminotransferase (OAT), and ornithine transcarbamylase (OTC) to yield putrescine, proline, and l-citrulline, respectively. Putrescine is sequentially converted into spermidine and spermine, through the sequential action of spermidine synthase (SDS) and spermine synthase (SMS) (green arrow).

Along the kynurenine pathway, three different enzymes catalyze the same rate-limiting step of Trp catabolism, i.e., IDO1, the IDO1 paralogue IDO2, and tryptophan 2,3-dioxygenase (TDO). The kynurenine pathway is initiated by the transformation of Trp into *N*-formylkynurenine that is rapidly converted into l-kynurenine (Kyn). The enzymatic cascade downstream of this metabolite generates several intermediates, collectivelly known as kynurenines, including 3-hydroxykynurenine (3-HK), 3-hydroxyanthranilic acid (3-HAA), and quinolinic acid (QUIN) (Figure 1). The final product of the pathway is the nicotinamide adenindinucleotide (NAD^+^) cofactor, which has a fundamental role in redox reactions that are essential for mitochondrial functions [13]. Kyn can also be transformed by kynurenine aminotransferase enzymes into kynurenic acid (KA), the end product of the lateral branch of the pathway. Kynurenines are endowed with several biological activities, such as immune regulation.

#### 2.1.1. IDO1

IDO1 represents one of the most interesting molecules that link an ancient metabolic pathway to immune regulation. IDO1 is a monomeric, heme-containing enzyme folded into a large domain containing the catalytic pocket and a small domain [14]. Besides transforming Trp into Kyn, IDO1 recognizes a broad variety of indole-containing substrates, including serotonin and melatonin [15]. IDO1 is expressed in many tissues and cells, including endothelial cells, fibroblasts, macrophages, myeloid derived suppressor cells (MDSCs) and DCs [16]. Normally expressed at low basal levels, it is rapidly induced by the cytokine interferon γ (IFN-γ) [17,18], mainly in DCs [19,20]. However, IDO1 does not only degrade Trp or produce kynurenines, but it does act as a signal-transducing molecule, an effect that leads to long-term expression of IDO1 in DCs and immune tolerance in vivo and is mostly independent on IDO1’s enzymic activity [21,22].

Several data have been reported for a possible association of IDO1 with RA pathogenesis. In sera of RA patients, Trp and Kyn concentrations were found to be reduced and increased, respectively, as compared to healthy controls [23,24]. Similarly, in another study, reduced baseline levels of Trp, 3-HK, and 3-HAA were accompanied by increased levels of Kyn and xanthurenic acid, indicating that the kynurenine pathway is highly active in RA patients [25]. However, significant differences in the levels of Trp, Kyn, or the ratio of Kyn to Trp (considered an indicator of systemic IDO1 activity [26]) between patients and control groups could not be found for all RA patients, suggesting the existence of a disease heterogeneity based on the kynurenine pathway [27].

The higher evidence of IDO1 in RA is in the synovial cavities. Synovial fibroblasts possess strong IDO1 activity [23,28] and suppress T cell responses through IDO1-mediated Trp depletion, which in turn activates the general control nonderepressible 2 (GCN2) kinase stress-response pathway and determines a down-regulation of the T cell receptor (TCR) ζ chain and cell-cycle arrest. Therefore, depletion of Trp by IDO1 in synovial fibroblasts is fundamental for the suppression of T helper (Th) cells and may act as a protective mechanism against inappropriate immune responses in non-arthritic joints [18]. In the synovial fluid (SF) of RA patients, but not healthy controls, KA was also detected. Moreover, its addition inhibited proliferation of synoviocytes in vitro, suggesting a regulatory role of KA in the control of the disease [29]. However, another study evidenced a down-regulation of Trp metabolites in the metabolomic profile of SF from RA patients as compared to matched controls [30]. Some authors explained this defect on the basis of aberrant methylation at the promoter of the cytotoxic T lymphocyte antigen 4 (CTLA-4) gene, which causes reduced regulatory T (Treg) cell function and loss of IDO1 activation, with a consequent impairment of the kynurenine pathway [31]. In addition, synovial tissues of RA patients are characterized by hypoxia [32], and hypoxic conditions are capable of reducing IDO1 expression, Trp metabolism, and T cell-suppressive capacities of synovial fibroblasts [33].

The protective role of IDO1 in joints and the impact of its defective expression and activity on the pathogenesis of RA have been demonstrated in several experimental models of RA. In the collagen-induced arthritis (CIA) murine model, IDO1 inhibition or deletion exacerbated the severity of arthritic symptoms and tissue damage [34]. IDO1-deficient mice presented increased numbers of T lymphocytes releasing IFN-γ and IL-17, especially in the joints, implying that IDO1 normally can suppress these cells [35]. In the same experimental model, Kolodziej et al. detected low Trp concentrations and an increase in Kyn in lymph nodes during the development of arthritis, indicating IDO1 activation and thus a possible pathogenetic role of the kynurenine pathway. In contrast, the downstream kynurenine metabolites anthranilic acid and 3-HAA accumulated during arthritis resolution, suggesting that they can play a protective role in this disease phase [36,37]. Consistent with this, Chen et al. found that arthritic symptoms are reduced by administering adenoviral vectors encoding IDO1 in CIA mice or by treatment with Kyn [38].

IDO1 is capable of exerting its protective role in RA also in the experimental model of antigen-induced arthritis (AIA), in which IDO1 can activate an anti-inflammatory program in plasmacytoid DCs (pDCs; a rare, yet functionally plastic DC subset) after IFN-α treatment [39]. In the same model, treatment with the soluble form of the CD83 molecule (sCD83) induced resolution of inflammation in an IDO1− and TGF-β−dependent manner, suggesting the involvement of IDO1 signaling activity [21]. In fact, sCD83 administration resulted in a long-term and antigen-specific immunomodulation in arthritis by upregulating IDO1 and TGF-β, reducing auto-aggressive effector T cells, inducing Treg cells, and promoting a direct impairment of osteoclastogenesis [40].

Overall, IDO1 seems to have a clear protective role in experimental models of arthritis and, at least in a subgroup, in RA patients. Therefore, the development of therapeutic approaches inducing IDO1 expression and activity may represent a good strategy for modulating immune responses in RA. In this regard, it could be helpful to stratify RA patients on the basis of *IDO1* single nucleotide polymorphisms (SNPs) [41,42] and the occurrence of a mutated form of IDO1 subjected to accelerated protein degradation and lower expression, as recently identified in a patient affected by multiple autoimmune disorders [43].

#### 2.1.2. IDO2

IDO2 is present in lower vertebrates and mammals and its expression is mainly confined to antigen-presenting immune cells, liver, kidney, brain, and placenta. The *IDO2* gene is located on chromosome 8 downstream of *IDO1* and the two genes have a similar genomic structure (43% amino acidic sequence similarity), suggesting that they may have derived from the duplication of a common ancestral gene, most likely more similar to the present IDO2. At variance with IDO1, for which a wide literature is available, the IDO2 function is still unclear [5].

The KRN strain is a spontaneous murine model of inflammatory autoimmune disease characterized by a rapid symmetrical onset of joint inflammation induced by the production of autoantibodies [44]. By using KRN mice and by performing adoptive transfer experiments on specific genetic backgrounds, IDO2 expression in B cells was found to be both necessary and sufficient to support robust arthritis development by acting at the T−B cell interface and thus modulating the potency of T cell help needed to promote autoantibody production [45,46]. A subsequent study also demonstrated that administration of a cell-penetrating anti-IDO2 monoclonal antibody significantly inhibits autoreactive T and B cell responses and alleviates joint inflammation, in both KRN and CIA models [47].

Thus, the studies conducted on the involvement of IDO2 in RA defined a key pathogenic role of the enzyme in the development of the pathology [48] and offered a proof of concept for antibody-mediated targeting of IDO2 as a new therapeutic strategy to treat RA.

#### 2.1.3. TDO

TDO, encoded by the *TDO2* gene, has similar high affinity for Trp as IDO1 but is characterized by a higher Trp specificity [49]. TDO displays 10% homology with IDO1, has a homotetrameric structure, and contains only the large catalytic domain. It is widely distributed in both eukaryotes and bacteria. In mammals, TDO is mainly expressed in the liver, but has also been found in mucous membranes, epididymis, and the brain [50]. TDO implication in RA and its effects on the immune system have yet to be determined. However, given that glucocorticoids are major inducers of TDO2, patients’ treatment with glucocorticoids could activate the kynurenine pathway via TDO2 in the liver, leading to the generation of several immunomodulatory metabolites. The fact that it receives massive amounts of Trp through the diet, liver TDO may be more responsible than IDO1 in regulating systemic levels of Trp and Kyn and thus inducing systemic immunosuppression.

#### 2.1.4. Kyn and the Aryl Hydrocarbon Receptor (AhR)

As mentioned above, Kyn represents the main product of IDO1, TDO, and possibly IDO2 (its affinity for Trp is 1000 lower than that of the other two enzymes [5] and therefore we do not know whether the production of Kyn represents its true catalytic mechanism). At variance with the majority of the other Trp metabolites produced along the kynurenine pathway, the molecular mode of action of Kyn is quite clear. In fact, Kyn has been proven to be an endogenous agonist of the aryl hydrocarbon receptor (AhR), a ligand-activated transcription factor [51]. Previously known as the receptor for 2,3,7,8-tetrachlorodibenzodioxin (TCDD), a highly toxic xenobiotic compound, AhR has been recently demonstrated to bind several other xenobiotics and endogenous ligands, which activate the receptor in a weaker, yet physiological manner [52,53]. AhR, expressed in an almost ubiquitous manner in human tissues and cells, is mainly involved in metabolic functions. However, its expression and activation in immune cells (either antigen-presenting cells, such as DCs, or T lymphocytes) translate into Treg cell-mediated immunoregulatory effects, which dampen immune responses, including inflammation [10,54]. Moreover, AhR and IDO1 may have coevolved to control exaggerated and autoimmune immune responses [55]. However, in the presence of specific ligands, such as 6-formylindolo[3,2-b]carbazole (FICZ; also a Trp metabolite), activation of AhR can instead promote the development of proinflammatory T helper 17 (Th17) cells.

Besides the indirect link via IDO1 and Kyn, some studies investigated the possible direct role of AhR in RA. For example, TCDD and other toxic compounds contained in cigarette smoke acted as AhR agonists, induced Th17 cells, and exacerbated the disease in a mouse experimental model of arthritis [56]. Incidentally, smoking is an important risk factor for RA. In RA patients, the induction of Th17 cells by cigarette smoke appeared to be mediated by AhR^+^ synovial DCs [57], probably characterized by an aberrant, proinflammatory profile that skews AhR activation towards Th17 instead of Treg cell development. In contrast to those data, accumulating evidences indicated that leflunomide, an immunomodulatory DMARD, is an agonist of AhR that, once activated, inhibits the expression of serum C-reactive protein (CRP) in hepatocytes [58]. Along this line, in macrophages from patients with RA but not osteoarthritis, AhR expression—and thus its immunoregulatory effect—is downregulated by high levels of miR-233, a microRNA (miRNA) [59].

Therefore, AhR, given its high promiscuity in terms of agonists and effects, appears to play a dual role, i.e., pro- and anti-inflammatory, depending on the ligand nature, cell expression, and presence of other signals in the cell microenvironment that may reprogram the functional profile of specific cells.

### 2.2. The Serotonin Pathway

Trp enters the serotonin pathway when the enzyme tryptophan hydroxylase (TPH) converts this essential amino acid into 5-hydroxytryptophan, which is then sequentially converted to serotonin (also known as 5-hydroxy-tryptamine (5-HT)), *N*-acetylserotonin (NAS), melatonin, plus other less characterized downstream metabolites (Figure 1). Besides being active as neurotransmitter (serotonin) or hormone/chronobiotic (melatonin), Trp metabolites produced along the serotonin pathway are endowed with anti-inflammatory and anti-oxidant effects via multiple biochemical mechanisms [60,61,62,63]. Thus, considering their safety and effectiveness, they are attracting widespread attention in a variety of diseases involving inflammation and oxidative conditions, including RA.

#### 2.2.1. Serotonin

Serotonin is a biogenic amine that activates seven types of receptors (six metabotropic, i.e., 5-HT1, 5-HT2, and 5-HT4-7, and one ionotropic, 5-HT3). In RA patients, increased levels of circulating serotonin, possibly released by activated platelets, have been reported. In the authors’ opinion, this finding may represent a negative predictor of bone mineral density in RA associated with a suppression of osteoblasts [64,65]. Serotonin levels are also elevated in RA SFs, where it induces synovial plasma extravasation via the release of inflammatory mediators [66,67]. Along this line, in mice models of arthritis, several studies reported the anti-inflammatory effect of 5-HT3 receptor antagonists [67,68]. Likewise, serotonin depletion by local injection of tropisetron, a 5-HT3 antagonist, attenuated disease severity, while intra-articular injection of serotonin caused joint inflammation and pain [69].

In the CIA model, an increased content of serotonin in the paws was reported. However, at variance with the studies described above, CIA mice deficient for TPH expression (*Tph1^−/−^*), and thus for serotonin production, were characterized by exacerbation of the clinical score, joint inflammation and erosion, bone resorption, osteoclast differentiation, release of pro-inflammatory factors, dampened expansion of Treg cells, and enhanced shift toward a proinflammatory Th17 phenotype, as compared to wild type CIA mice [70]. Consistent with this, a correlation between the severity of the disease and a lower density of a serotonin receptor (5-HT2A) was described in a cohort of patients with RA. Furthermore, a genetic polymorphism of the *5HT2A* gene associated with susceptibility for RA and modulation of the proinflammatory cytokine response of T cells and monocytes [71,72,73]. Moreover, administration of 5-hydroxytryptophan, an intermediate in serotonin synthesis, ameliorated arthritis in the CIA model by suppressing the production of proinflammatory cytokines and increasing serotonin levels [74].

Because serotonin is a neurotransmitter, several authors investigated if and how peripheral inflammation in RA could have a role in the modulation of neuronal activity. Brown et al. described that, in CIA mice, serotonin levels are altered not only in peripheral tissues but also in the brain, especially in the hippocampus. They found that upregulation of the serotonin transporter (SERT) activity and enhancement of serotonin reuptake from the synapse can contribute to the development of anhedonia [75]. In other studies, IDO1 upregulation in RA was found to be responsible for the decrease in the serotonin/Trp ratio in the hippocampus of mice suffering from arthritis [76,77]. Moreover, the existence of a link between inflammation and immune alterations in RA and depression has been hypothesized, suggesting that targeting immunological pathways could treat not only RA but also alleviate mental health burden [78]. Conversely, selective serotonin re-uptake inhibitors (SSRIs), the first therapeutic choice in depression, can affect arthritis development. Indeed, by blocking serotonin re-uptake, SSRIs exert central effects by increasing serotonin levels in the brain, thus improving depression, and also reduce disease severity in RA patients [79], as well as decrease arthritis scores in CIA mice by suppressing cytokine production from macrophages and synovial membrane cells [80]. However, a recent study indicated that the use of SSRIs is related to a higher risk of fractures in patients with RA [81].

Overall, the available data confirm the multifaceted activity of serotonin, possibly due to the high number of 5-HT receptors and the variety of effects triggered by each of them in distinct tissues and cells.

#### 2.2.2. NAS

NAS is produced from serotonin by the arylalkylamine N-acetyltransferase (AANAT) enzyme along the serotonin pathway (Figure 1). Although NAS is predominantly synthesized in the pineal gland, a series of studies provided experimental evidence that NAS and AANAT activity are also present in the hippocampus, the olfactory bulb, the spinal cord, and the cerebellum [82]. Previously considered to be simply an intermediate metabolite between the serotonin and melatonin, NAS has recently been shown to exert significant anti-inflammatory and protective effects in a mouse experimental model of multiple sclerosis, a chronic inflammatory/autoimmune disease, via direct binding of IDO1 and enhancement of its catalytic activity in DCs [42]. However, the role of NAS in RA has not been investigated so far. Thus, in light of its anti-inflammatory and antioxidant effects and considering the role played in RA by other metabolites belonging to the same metabolic pathway, the possible involvement or therapeutic efficacy of NAS in RA should be investigated.

#### 2.2.3. Melatonin

Melatonin is synthesized from NAS by hydroxyndole-O-methytransferase (HIOMT), mainly in the pineal gland but also in several extrapineal organs, including the brain, retina, gastrointestinal tract, bone marrow, lymphocytes, and skin [63]. Melatonin is one of the major neuroendocrine hormones that show a remarkable functional versatility. Indeed, although its primary function consists of the regulation of circadian rhythm, melatonin also promotes mitochondrial homeostasis, proliferation, apoptosis, metastasis, oncostatic, anti-aging, antioxidant, and immunomodulatory properties [62,63,83].

Melatonin is beneficial in several inflammatory autoimmune diseases, including systemic lupus erythematosus, multiple sclerosis, inflammatory bowel disease, and type 1 diabetes [62]. However, its effects in RA remain controversial. In the CIA model, the development of arthritis was exacerbated by constant darkness (which induces high physiological levels of melatonin) [84] and by daily administration of melatonin [85]. In the same model, pinealectomized mice (characterized by a 70% reduced level of melatonin in sera as compared to controls) showed a reduced severity of arthritis [86]. Interestingly, melatonin administration increased the severity of arthritis by decreasing the expression of cryptochrome1 (*Cry1*), a circadian clock gene, and by increasing serum concentrations of IL-6 and TNF-α, two pro-inflammatory cytokines [87]. Altogether, these evidences suggested that melatonin is pathogenetic in the CIA mouse model. However, in other studies conducted in the same animal model, melatonin appeared to possess a dual effect, i.e., as a pro-inflammatory agent and antioxidant [88], and also appeared to exert a beneficial effect by reducing paw swelling, bone erosion, and cartilage degradation via inhibition of TNF-α and IL-1β production [89]. Similarly, administration of melatonin in the AIA model in rats inhibited the inflammatory response [90].

In RA patients, melatonin levels in sera were found to be significantly higher than healthy subjects, and these levels correlated positively with disease activity scores and the erythrocyte sedimentation rate (ESR) [91]. Moreover, in the early morning when melatonin levels are higher, patients with RA exhibited high serum concentrations of pro-inflammatory cytokines, especially TNF-α and IL-6 [92,93]. However, other studies reported that serum melatonin levels in the morning do not relate with disease activity in RA patients, despite observing that newly diagnosed RA patients have higher serum melatonin values [94]. In addition, studies on synovial tissue specimens from RA patients and cultures of human rheumatoid fibroblast-like synoviocytes demonstrated that melatonin exerts anti-inflammatory activities by downregulating TNF-α and IL-1 production via suppression of PI3K/AKT, ERK, and NF-κB signaling and upregulation of miR-3150a-3p (a microRNA) expression, in an MT1 receptor-dependent manner [89]. Notably, SNPs in the gene coding for melatonin receptor type 1B (*MTNRIB*) have been associated with the presence of the rheumatoid factor (RF) in RA patients [95].

Therefore, on one hand, several researches suggest that melatonin may enhance pro-inflammatory activities and promote disease activity in RA, while, on the other, other preclinical and clinical investigations document substantial anti-inflammatory and immunoregulatory properties of melatonin in the same disease [83]. Some investigators have suggested that melatonin could be effective as adjunctive therapy in the treatment of RA [96]. However, more studies are certainly needed to safely pursue this direction.

## 3. Arginine Metabolism and RA

Arg is a basic amino acid classified as conditionally essential. In fact, although endogenously synthesized, Arg may become limited in the case of catabolic stress and pathologic conditions (including infections, trauma, and cancer), making exogenous supplements necessary. Arg deprivation can profoundly suppress T cell responses [8,97] by decreasing cyclin D mRNA translation and blocking the activity of several cyclin-dependent kinases, which are essential for the cell cycle advancement from G_0_/G_1_ to S-phase, as well as promoting T-cell anergy/paralysis via the down-regulation of the TCR ζ chain [98].

Arg is the common substrate of three isoforms of nitric oxide synthases (NOS1−3) and two arginases (ARG1 and ARG2) (Figure 1) [99]. NOS enzymes transform Arg into nitric oxide (NO) and l-citrulline, whereas arginases catalyze the conversion of Arg into l-ornithine (Orn) and urea. The two pathways, namely, that mediated by the NOS2 enzyme and that mediated by ARG1, are among the main functional features of M1 and M2 macrophage subsets, respectively. M1 cells exploit NO production for “killing/fighting” purposes and thus inflammatory responses, whereas ARG1 activity in M2 cells mainly leads to Arg starvation and thus immunoregulatory and “healing/fixing” effects [100]. Of note, Orn can be further transformed by ornithine decarboxylase (ODC) into polyamines (Figure 1), i.e., biogenic amines endowed with several biologic effects. Arg can also be methylated and transformed into l-*N*^G^-monomethyl arginine (MMA), asymmetric dimethylarginine (ADMA), and symmetric dimethylarginine (SDMA) [101], which are emerging as biomarkers of cardiovascular complications in diabetes mellitus [102].

Given such versatility and its importance in health and disease [103], Arg metabolism has high chances of being implicated in the molecular mechanisms of RA pathogenesis, as outlined below.

### 3.1. ARG1 and ARG2

Mammalian arginases, ARG1 and ARG2, catalyze the same biochemical reaction but are distinguished by different cellular expression, molecular regulation, and subcellular localization [104,105]. ARG1 is a constitutive cytosolic enzyme mainly expressed in the liver, erythrocytes, and human polymorphonuclear neutrophils. In mice, ARG1 is also present in other immune cells, such as group 2 innate lymphoid cells, macrophages, and DCs. ARG1 is upregulated in murine myeloid cells (mainly M2 macrophages and MDSCs) by Th2 cytokines, such as IL-4 and IL-10, and also TGF-β, with consequent induction of fibrosis and tissue regeneration [106,107]. While significant interspecies differences exist, arginase-mediated immunopathology appears to be comparable between species.

Several investigations have reported significant alterations of arginase enzymes in RA patients. In one study, arginase activity was measured in SF cells from patients with different forms of arthritis (RA, osteoarthritis, psoriasis, arthralgia, and juvenile chronic arthritis), either directly or after in vitro stimulation with inflammatory agents [108]. The authors found that SF cells constitutively express ARG2, which could be further upregulated by lipopolysaccharide. Overexpression of ARG2 inhibited the production of NO by substrate competition, even in the presence of IFN-γ, a potent NOS2 inducer [108]. In contrast to SF cells, fibroblasts from the synovial membrane expressed NOS2 and released NO to the culture medium. This difference between cell types in the ability to generate NO was explained as a difference in cytokine profiles in the tissue versus SF. In another study, arginase activity was measured in the plasma of RA patients. The results showed an increased level of plasma arginase activity, as well as decreased Arg bioavailability and increased plasma concentrations of methylated metabolites of Arg, i.e., asymmetric dimethylarginine (ADMA) and symmetric dimethylarginine (SDMA) (see below).

Because increased arginase activity reduces the production of the vasodilator NO, dysregulated Arg metabolism may represent a risk factor for cardiovascular diseases, whose incidence is indeed increased in RA patients [109]. In rats with AIA, Prati et al. investigated whether the upregulation of arginases, which reciprocally regulates the endothelial NOS isoform (i.e., NOS3) and thus the production of NO, may contribute to the endothelial dysfunction typical of the disease. They found that AIA rats exhibit higher expression and activity of both ARG1 and ARG2 in the endothelium and that incubation of aortic rings of AIA rats with nor-NOHA, an arginase inhibitor, enhances the vascular response to acetylcholine [110]. In this regard, it might be interesting to note that although methotrexate (MTX; a first-line DMARD used for decades) increases NOS activity, it does not improve the endothelial function in AIA rats, and this may be correlated with the inability of MTX to interfere with arginase expression and activity [111]. In contrast, treatment with infliximab, a chimeric monoclonal antibody neutralizing the pro-inflammatory cytokine TNF-α (considered a DMARD) did decrease plasma arginase activity in at least 40% of RA patients. However, this effect was correlated with a parallel increase in Th1/Th17 inflammatory cytokines by peripheral blood mononuclear cells (PBMCs), interpreted as a consequence of reduced arginase-dependent immunosuppressive effects [112].

A positive role of arginase enzymes in RA was recently proposed by a study aimed at investigating the function of the Fos-related antigen 1 (Fra-1) during macrophage activation and development of arthritis [113]. Fra-1 is a transcription factor that, when expressed in alveolar macrophages, mediates the inflammation lung injury induced by lipopolysaccharide [114]. By using the K/BxN arthritis mouse model and mice lacking Fra-1 expression selectively in macrophages, the authors found that Fra-1 enhances the clinical course of the disease and that this occurs via repression of the *Arg1* gene [113]. Moreover, patients with active RA showed increased Fra-1 expression in the peripheral blood and elevated Fra-1 protein in synovial macrophages as compared with RA patients in remission. In addition, the Fra-1/ARG1 ratio in synovial macrophages correlated with RA disease activity [113]. However, although the authors thoroughly investigated the inflammatory signs of RA, which can indeed be counteracted by ARG1, the possible pathogenetic outcome of ARG1 (hypothesized by others) on the endothelial vascular tone was not addressed.

Thus, overall, the available data would indicate that arginase enzymes are overexpressed in RA. On one hand, ARG1/2 overexpression in immune cells, such as macrophages, might represent an attempt to contain inflammation/autoimmunity. On the other hand, the increased consumption of Arg by ARG1/2 may significantly reduce the levels of NO, the main vasodilator of the organism, thus increasing the risk of cardiovascular diseases associated with RA.

### 3.2. ODC and Polyamines

Polyamines, such as putrescine, spermidine, and spermine, are polycationic molecules present in almost all living cells and are integral to a wide range of biological functions, including gene transcription and translation, cell growth, and death [115,116]. Such pleiotropic nature relies on the polyamines’ ability to bind negatively charged macromolecules, i.e., nucleic acids, proteins, and phospholipids [117]. Polyamines are produced downstream of ARG1/ARG2. In fact, Orn, one of the arginase products, is the substrate of ODC that, by decarboxylation, produces putrescine, the precursor of spermidine, which in turn is the precursor of spermine.

RA patients have been found to accumulate spermine and spermidine in synovial tissue, SF, PBMCs, and urine. Inflammatory cytokines and other stimuli can induce the increase in polyamines in RA [118]. Polyamines can be converted by monocytes into toxic compounds, including hydrogen peroxidase and ammonia [119]. It has been hypothesized that MTX, by inhibiting dihydrofolate reductase, may reduce the availability of methyl donors and thus the generation of polyamines. However, this does not seem to be the major therapeutic mechanism of MTX in RA.

Other studies indicated that polyamines could instead be beneficial for RA. In fact, spermine ameliorated the destruction of cartilage and bone in the joints of CIA rats [120]. Moreover, both spermidine and spermine inhibited osteoclast differentiation in vitro and prevented bone loss in vivo in ovariectomized mice [121], an effect possibly due to the inhibition of migration of pre-osteoclasts during the fusion step [122]. Along the same line, ARG1 (upstream polyamine production) and NO have been found to play reciprocal roles as negative and positive regulators, respectively, of osteoclast differentiation [123].

Therefore, similar to ARG1 and ARG2, abnormal levels of polyamines can be observed in RA. However, it is still unclear whether this increase is a pathogenetic or counteracting mechanism of the disease.

### 3.3. The NOS Pathway

The three NOS isozymes catalyze the same reaction but have different distribution and regulation. NOS1 (also known as neuronal) and NOS3 (the endothelial form) are mostly constitutive enzymes, whereas NOS2 (inducible NOS) is upregulated mainly in immunocytes by pro-inflammatory cytokines and microbial products. All NOS generate NO, a short-lived gas that diffuses through lipid membranes and acts as a signaling molecule via activation of soluble guanylate cyclase. In inflammatory conditions, inducible NOS2 can be greatly upregulated and thus produce high levels of NO that, being a radical, exerts toxic effects on cells of the microenvironment. Because Arg is the sole precursor of NO and ARG1/ARG2 are upregulated in RA in both SF and plasma, NO levels should be generally reduced in patients with RA. Furthermore, because NOS enzymes use O_2_ to produce NO, the low level of Arg caused by upregulated arginase activity may promote NOS uncoupling and consequent production of superoxide anion with toxic outcomes [124]. However, several studies showed instead an increased expression of NOS2 in RA patients [125], particularly in PBMCs, which correlates with disease activity [126].

As mentioned above, some studies indicated NO-favoring osteoclast differentiation [123]. However, this issue appears to be more complex. In fact, data obtained by Nilforoushan et al. indicated that NO may have a stage-dependent, biphasic effect on osteoclast differentiation. Initially, NO would regulate negatively pre-osteoclast differentiation but, later, would facilitate the fusion of pre-osteoclasts by up-regulating actin remodeling [127]. The different effects of NO on pre-osteoclast differentiation may depend on its concentration. Low concentrations of NO would enhance osteoclast generation, whereas higher concentrations inhibit osteoclast generation and activity [128].

Again, opposite findings and outcomes have been observed for NO, the NOS product, in RA. On one hand, high levels of NO may translate well into inflammatory and thus injury effects, although it may also inhibit pre-osteoclast differentiation and thus protect from progressive bone loss. On the other hand, low, yet physiological concentrations of NO at the vascular level may protect from endothelial dysfunction and thus cardiovascular diseases often associated with RA.

### 3.4. Methylated Arg Products

Although much less is known as compared to the other Arg metabolites, methylated Arg derivatives are emerging as important biologic modulators. For example, MMA and ADMA are potent endogenous inhibitors of NOS, whereas SDMA inhibits NO production by blocking the cellular uptake of Arg [101]. These effects can lead to vascular dysfunction. To date, a de novo synthetic pathway of N-monomethylarginine (NMMA), ADMA, or SDMA from free Arg has not been documented. Therefore, free methylarginines found in plasma and cells would derive solely from protein turnover and degradation of proteins containing methylarginines [129]. By using a comprehensive metabolomic analysis, significantly higher levels of ADMA and SDMA were found in the plasma of RA patients as compared to healthy controls [109]. More specifically, plasma SDMA was associated with hypertension and hyperlipidemia in patients with RA [109], possibly suggesting this metabolite being a biomarker of cardiovascular risk in RA.

## 4. Amino Acid Metabolism by Microbiota and RA

The immune system is continuously exposed to a series of microorganisms—bacteria, fungi, helminths, and viruses—mainly resident in the gut and termed microbiota. The immunity-microbiota interaction has evolved to favor tolerance and co-dependence between host and microbes [130]. However, a combination of environmental factors and genetic defects can result in the break of immune tolerance and intestinal homeostasis [46]. Therefore, the microbiota is considered to play an important role in the pathogenesis of autoimmune diseases, including those outside the gut. In fact, this effect can be explained by either migration of pro-inflammatory T helper subsets, such as Th17 cells [131], from the gut to other organs or the production of metabolites from the microbiota that can diffuse systemically [132]. These metabolites can be pro-inflammatory, and thus be initiators of the disease, or may serve as immune regulators, and thus be protective. Therefore, the identification of such compounds may provide opportunities to control autoimmunity and thus RA, although we still do not know whether the composition of gut microbiota is the cause or the result of a specific disease status [133]. Relevant compounds produced by the microbiota for the maintenance of immune homeostasis include, but are not limited to, short-chain fatty acids, Trp metabolites, and polyamines.

### 4.1. Trp Metabolites

Regarding Trp metabolites, the gut microbiota can produce several molecules, some of which are not produced by the host but can nevertheless activate AhR and thus induce immunoregulatory effects [134]. These include indole-3-aldehyde (Iald), indole-3-acetic acid (IAA), 3-methylindole, and indole-3-lactic acid (ILA) [135]. The production of indoxyl-3-sulfate (also an AhR agonist) from Trp is achieved by the cooperative metabolism of tryptophanase-positive bacteria and the host [136]. This kind of molecule is known as “co-metabolite”, i.e., produced from the interplay between microbiota and host metabolisms [137,138].

#### 4.1.1. Iald

In the gastrointestinal tract, diet-derived AhR ligands promote local IL-22 production by innate lymphoid cells (ILCs), a population now referred to as group 3 ILCs (ILC3s) [139]. Iald, representing one of these ligands and mainly produced by lactobacilli, can indeed balance mucosal reactivity in the gut via ILC3s producing IL-22 [11]. Moreover, ILCs induced by Iald upregulate the expression of β-defensin 14 on pancreatic β-cells and protect from autoimmune diabetes [140]. Although the effects of Iald in inflammatory/autoimmune disease are very promising, no significant study on Iald has been performed in rheumatic diseases yet.

#### 4.1.2. IAA

Recent evidences have linked IAA with the resistance to liver diseases, including those induced by high-fat diet (nonalcoholic fatty liver disease). The effect is associated with amelioration in insulin resistance, lipid metabolism, and inflammatory stress [141]. In fact, IAA reduces the number of F4/80^+^ macrophages infiltrating the liver and the expression of monocyte chemoattractant protein 1 (MCP-1) and TNF-α. Moreover, IAA production is reduced both locally in the gut and systemically in patients with inflammatory bowel diseases [142]. Although no direct evidence for a protective effect of IAA in RA has been provided yet, decreased bacterial diversity characterizes the altered gut microbiota in patients with psoriatic arthritis, resembling dysbiosis in inflammatory bowel disease and thus a possible deficiency in IAA [143]. Quite interestingly, a high-salt diet, widely considered to cause health problems (gut microecological imbalances, constipation, and hypertension), also significantly reduces IAA production, because cells of *Lactobacillus murinus*, the main provider of the Trp metabolite, are suppressed. Conversely, administration of IAA^+^ *L. murinus* cells protects from experimental autoimmune encephalomyelitis (EAE; an experimental model of multiple sclerosis) by reducing proinflammatory Th17 cells [144].

#### 4.1.3. ILA

Numerous studies have explored the therapeutic effects of human umbilical mesenchymal stem cells (HUMSC) that mediate the balance between Treg and Th17 cells in RA [145]. In a very recent study, human umbilical mesenchymal stem cells were transplanted in rats with CIA. Results showed that HUMSC transplantation reduced the pathology scores and the degree of bone damage in the ankles, favored the induction of Treg versus Th17 cells, increased the genera *Bacteroides* and *Bacillus*, and consistently upregulated ILA levels in plasma and AhR activation in the ileum [146]. The production of Trp metabolites, both in quality and quantity, is not the same among distinct bacteria strains. For example, Bifidobacterium strains, most of which are highly present in infants, produce only ILA as an AhR agonist [147]. Therefore, alterations in the composition of microbiota may greatly influence the overall Trp metabolism in the gut that can translate into immune dysregulation.

### 4.2. Arg Metabolites

Polyamines are also considered co-metabolites and their alteration in the gut can induce either toxic effects, cancer [137], or enhance the development and maintenance of the intestinal mucosa (particularly spermidine) and resident immune cells [148]. In a rat model of spondyloarthritis, administration of short-chain fatty acids attenuated the disease and upregulated the production of spermidine in cecal contents [149]. However, whether this increase may be involved in the protective effect has been unclear.

### 4.3. Dysbiosis, Amino Acid Metabolism, and RA

Metagenomic analyses recently demonstrated that the oral and gut microbiomes are perturbed in RA and partly normalized after treatment [150]. In addition, the chronic oral inflammation caused by oral bacteria and leucocyte infiltration with progressive destruction of the alveolar bone seems to share the same pathogenetic mechanisms with RA [151]. Therefore, in RA, an altered microbiota appears to be a likely candidate as an environmental risk factor, as well as a pharmacological target_._ In this regard, the therapeutic effect of an “old” DMARD, such as sulfasalazine, may be related to a bactericidal activity, at least in some patients [152].

Therefore, although much work is still needed to mechanistically link microbiota-derived metabolites and co-metabolites with molecular and cellular alterations in RA, promising results have already been obtained. In a very recent study, butyrate (a short-chain fatty acid) supplementation reduced experimental arthritis severity via an increase in 5-hydroxyindole-3-acetic acid, a Trp metabolite derived from serotonin and agonist of AhR [153]. The activation of such a mechanism promoted the differentiation of B lymphocytes into regulatory cells. These data demonstrated that supplementing the diet with certain microbiota-derived molecules, which directly or indirectly favor AhR activation by Trp metabolites, may be a promising treatment for RA [153].

## 5. Conclusions

**Figure 2:**
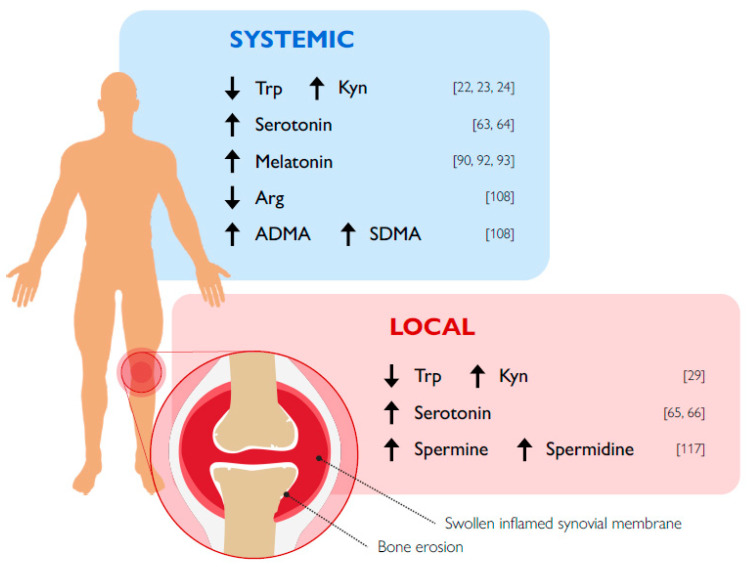
Schematic representation of significant changes in tryptophan and arginine metabolite levels in RA patients. Numbers on the rights represent relevant references.

In RA, despite the availability of several DMARDs, there is still the need to develop new therapeutic options that would not only possess fewer side effects but ideally cure the disease. The emerging field of immunometabolism seems to represent a possible source for novel therapeutic strategies in several chronic inflammatory/autoimmune diseases, such as RA. In the current review, we focused our analysis on the known role of Trp and Arg metabolisms, representing critical immune checkpoint mechanisms in health and neoplasia, in the pathogenesis of RA (Figure 2). In general, a greater number of studies in experimental models of arthritis was performed for Trp, as compared to Arg metabolism, and indicated that at least the IDO1-mediated kynurenine pathway clearly exerts protective effects. If more variable results had been obtained in RA patients, an occurrence would possibly be due to the heterogeneity of the disease. In the case of Arg metabolism, because ARG1/2, polyamines, and NOS are strictly correlated, it was difficult to determine the net role of each of them in RA. Nevertheless, the high activity of ARG1/2, particularly at the synovial level, seemed to be a common feature of RA patients. Therefore, on one hand, this should favor an increase in multitasking polyamines and, on the other, NO production should be reduced, an event possibly translating in cardiovascular complications, often observed in RA patients. A further level of complexity appeared to be determined by the fact that NO effects depend on both its concentration, which can vary in a range of more than three orders of magnitude, and the kinetics of production.

In conclusion, although no uniform picture emerged, the bulk of data indicated the kynurenine pathway rather than Arg metabolism as a suitable target to be potentiated in RA. Nevertheless, further investigations in Arg metabolic pathways in RA may provide important pieces of information to better define its biology and pathogenetic meaning.
